# Synthesis of Poly(norbornene-methylamine), a Biomimetic of Chitosan, by Ring-Opening Metathesis Polymerization (ROMP)

**DOI:** 10.3390/md15070223

**Published:** 2017-07-14

**Authors:** Na Li, Huanhuan Wang, Xiaosai Qu, Yu Chen

**Affiliations:** School of Materials Science & Engineering, Beijing Institute of Technology, Beijing 100081, China; bitna_li@foxmail.com (N.L.); Wangh208@126.com (H.W.); shawnqu@foxmail.com (X.Q.)

**Keywords:** ROMP, poly(norbornene-methylamine), controllable, living polymerization, chitosan

## Abstract

ROMP is an effective method for preparing functional polymers due to its having characteristics of “living” polymerization and rapid development of catalysts. In the present work, poly(norbornene-methylamine), a mimic of chitosan, was synthesized via ROMP reaction. The amino-protected product, 5-norbornene-2-(*N*-methyl)-phthalimide, was prepared by a reaction of 5-norbornene-2-methylamine with phthalic anhydride, which was then subjected to the ROMP reaction in the presence of Hoveyda-Grubbs 2nd catalyst to afford poly(norbornene-(*N*-methyl)-phthalimide). The target product, poly(norbornene-methylamine), was obtained by deprotection reaction of poly(norbornene-(*N*-methyl)-phthalimide). The products in each step were characterized by FTIR and ^1^H-NMR, and their thermal stabilities were determined by TG analysis. The effects of molar ratio between monomer ([M]/[I]) and catalyst on the average relative molecular weight ($\bar{Mn}$) and molecular weight distribution of the produced polymer products were determined by gel permeation chromatography (GPC). It was found that the $\bar{Mn}$ of poly(norbornene-(*N*-methyl)-phthalimide) was controllable and exhibited a narrow polydispersity index (PDI) (~1.10). The synthesis condition of 5-norbornene-2-(*N*-methyl)-phthalimide was optimized by determining the yields at different reaction temperatures and reaction times. The highest yield was obtained at a reaction temperature of 130 °C and a reaction time of 20 min. Our work provides a new strategy to synthesize polymers with controllable structures and free –NH_2_ groups via ROMP.

## 1. Introduction

**Scheme 1:**
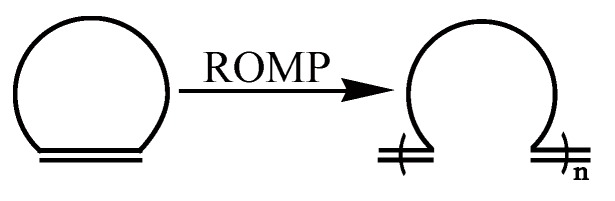
The reaction mechanism of ROMP.

ROMP has been an effective method for preparing functional polymers due to its having the characteristics of living polymerization [1,2] and rapid development of catalysts [3,4,5,6]. The mechanism of ROMP is substantially different from other polymerization reactions, such as radical polymerization and anionic polymerization. During an ROMP reaction, the double bond translocates continuously, the chain gradually grows, and the double bond of monomer molecules remains in the generated polymer molecules as shown in Scheme 1 [7,8]. The resultant polymer of ROMP can inherit the single bond, double bond and even the ring structure of a double-ring monomer. Therefore, ROMP can be used to prepare completely alternating copolymer [9,10]. In addition, its advantages of mild reaction conditions, high reaction rate, absence of chain transfer and termination reaction in most cases provide the polymers a narrow molecular weight distribution. Over the last few decades, many new controllable polymers, such as new homopolymers, telechelic copolymers [11,12,13], block copolymers [14,15,16], graft copolymers [17,18,19], liquid crystal polymers [20], and so on, have been synthesized by ROMP. These novel polymers have been extensively employed as conductive materials, porous network materials, self-healing materials and biomedical materials.

Norbornene (NBE) and its derivatives are highly reactive due to their special cyclic structure, and thus are prone to a variety of polymerization reactions, such as radical polymerization, anionic polymerization, vinyl addition polymerization [21], and ROMP, in the presence of catalysts. Wherein, ROMP is one of the most commonly used polymerization methods due to its special reaction mechanism and mild reaction conditions. The ROMP of boron-, fluorine-, chlorine-, nitrile-, silicon-, silicon-, oxygen-, amides- and acid anhydride- derivatives of NBE has been well studied [22,23,24,25,26,27]. However, the ROMP of amino NBE has rarely been reported. Similar to chitosan, the cyclic polymers bearing –NH_2_ usually possess excellent hydrophilicity, high reactivity and excellent complexing abilities with metal ions [28], and thus they can be applied in a variety of fields. In addition, the –NH_2_ can be converted to –NH_3_^+^ in acidic conditions, which provides a pH sensitivity to the polymers. Therefore, these polymers are promising candidate materials for gene transfection vectors and drug release.

**Scheme 2:**
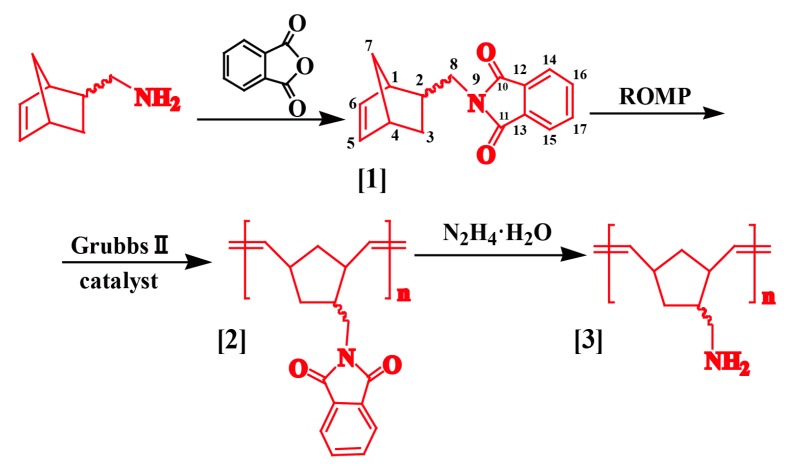
The reaction route for preparing poly(norbornene-methylamine) by ROMP.

In the present work, poly(norbornene-(*N*-methyl)-phthalimide) was synthesized by the ROMP of 5-norbornene-2-(*N*-methyl)-phthalimide, an amino-protected product of 5-norbornene-2-methylamine. Its anhydride group was then deprotected to afford poly(norbornene-methylamine). The synthesis route is shown in Scheme 2. Our work provides a new strategy for the synthesis of polymers bearing –NH_2_ groups with controllable structures by ROMP. The intermediate product, poly(norbornene-(*N*-methyl)-phthalimide), can be used to prepare block copolymers with other unsaturated compounds. It also can be considered as a mimetic of natural chitosan with appropriate modifications. The produced polymer can be potentially applied in a variety of fields, such as gene transfection vectors, controlled-release carriers for functional drugs, and so on.

## 2. Results and Discussion

### 2.1. FTIR Characterization

**Figure 1:**
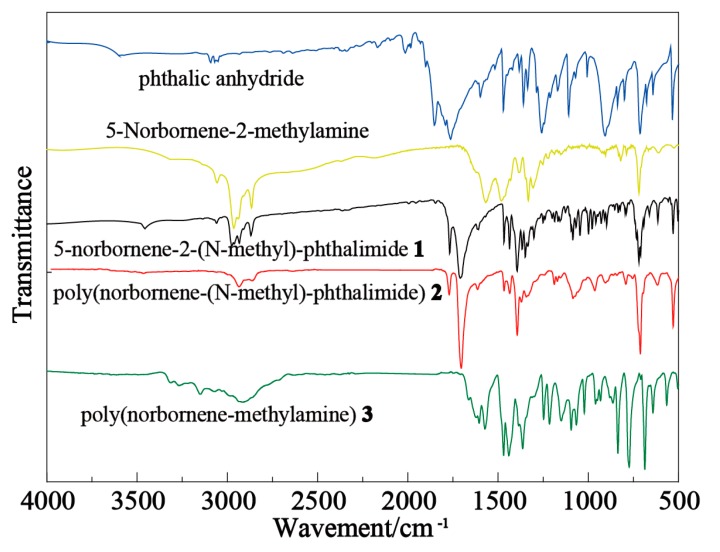
FTIR spectra of raw materials and synthesized products.

A comparison between the FTIR spectra of phthalic anhydride and 5-norbornene-2-(*N*-methyl)-phthalimide (product **1**) indicates that the symmetric and asymmetric stretching vibration absorption peaks of C=O groups shifted from 1762 cm^−1^ and 1855 cm^−1^ (phthalic anhydride) to 1709 cm^−1^ and 1768 cm^−1^ (5-norbornene-2-(*N*-ethyl)-phthalimide), respectively, due to the formation of a lactam group between the –NH_2_ group and C–O–C group (Figure 1). The N-H stretch vibration absorption peaks of the –NH_2_ in 5-norbornene-2-methylamine at 1573 cm^−1^, 1483 cm^−1^ and 3330 cm^−1^ disappeared after the reaction with phthalic anhydride, indicating the formation of phthalimide group. The peak at 3000 cm^−1^ was attributed to the stretching vibration of –CH_2_– groups of product **1**. The peaks at 2860 cm^−1^ and 3062 cm^−1^ were due to the stretching vibration of the unsaturated =CH group. 

Poly(norbornene-(*N*-methyl)-phthalimide) (Product **2**) exhibited a similar FTIR spectrum to that of product **1**, except that the stretching vibration peak of =CH at 3060 cm^−1^ disappeared due to the ring-opening reaction.

As product **2** was converted to poly(norbornene-methylamine) (Product **3**), the peak at 1716 cm^−1^ that was attributed to the C=O group of phthalic anhydride disappeared, and new bands appeared at 3324 cm^−1^, 3276 cm^−1^, 1608 cm^−1^, 1573 cm^−1^ and 762 cm^−1^. The bands at 3324 cm^−1^ and 3276 cm^−1^ can be assigned to the symmetric and antisymmetric stretching vibrations of –NH_2_ groups, respectively. The bands at 1608 cm^−1^ and 1573 cm^−1^ were due to the in-plane bending vibrations of N-H group and that at 762 cm^−1^ was ascribed to the out-plane wagging vibrations of N-H group. These results indicate that target compounds were successfully synthesized and exhibited the expected structures.

### 2.2. ^1^H-NMR Characterization

**Figure 2:**
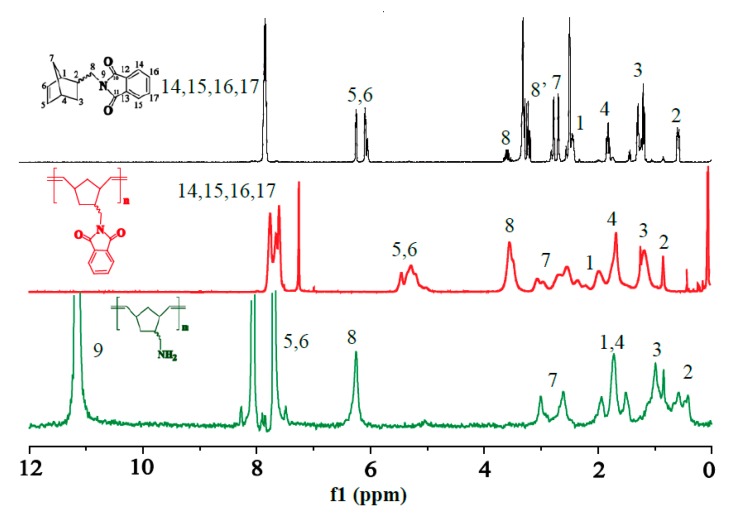
^1^H-NMR spectra of products **1**, **2** and **3**.

The ^1^H-NMR spectra of the products in each step are shown in Figure 2. The chemical shift at 7.9 ppm of product **1** was ascribed to the 14H, 15H, 16H and 17H on the phenyl ring and the peak at 6.1–6.3 ppm was caused by the 5H and 6H on the NBE ring. The peaks at 3.5–3.6 ppm and 3.2–3.3 ppm were due to the 8H on -(*N*-methyl)-phthalimide, and the peak at 2.7–2.9 ppm was assigned to the 7H on the NBE ring bridge. The peaks at 2.4–2.5 ppm and 1.7–1.9 ppm were attributed to the 1H and 4H on the NBE ring, respectively. The 3H and 2H on the NBE ring caused the chemical shifts at 1.2–1.4 ppm and 0.5–0.6 ppm, respectively. 

No significant differences between the ^1^H-NMR spectra of products **1** and **2** were observed, except that the peak at 6.1–6.3 ppm of product **1** shifted to 5.1–5.6 ppm in the spectrum of product **2**. The shift might be attributed to the transformation of H on the ring double bond into the H of double bond on the main chain during the ring-opening reaction of the NBE derivative. The chemical shift at 7.5–7.9 ppm of product **2** was ascribed to the 14H, 15H, 16H and 17H on its phenyl ring. The peaks at 3.3–3.7 ppm was due to the 8H on -(*N*-methyl)-phthalimide and the peak at 2.4–3.2 ppm could be assigned to the 7H on NBE ring bridge. The peaks at 1.8–2.3 ppm were ascribed to the 1H on the NBE ring and those at 1.6–1.8 ppm were attributed to the 4H on the NBE ring and the H of D_2_O in the CDCl_3_ solvent. The chemical shifts at 1.1–1.4 ppm and 0.8–0.9 ppm could be assigned to the 3H and 2H on the NBE ring.

Product **3** exhibited a significantly different ^1^H-NMR spectrum from product **2** due to the deprotection of phthalic anhydride. The –NH_2_ group obtained from the removal of phthalic anhydride exhibited an H peak at 11.0–11.1 ppm. The peak at 5.1–5.6 ppm of product **2** shifted to 7.6–8.3 ppm in the spectrum of product **3**, possibly because the free –NH_2_ group is attached to a double bond on the main chain. The peaks at 6.2–6.4 ppm and 2.6–3.1 ppm were assigned to the 8H on -(*N*-methyl)-phthalimide and the 7H on the NBE ring bridge, respectively. The chemical shift at 1.4–2.1 ppm was attributed to the 1H and 4H on the NBE ring. The peaks at 0.7–1.2 ppm and 0.4–0.7 ppm were ascribed to the 3H and 2H on the NBE ring, respectively.

These ^1^H-NMR data indicate that the target products were successfully synthesized.

### 2.3. TG Characterization

**Figure 3:**
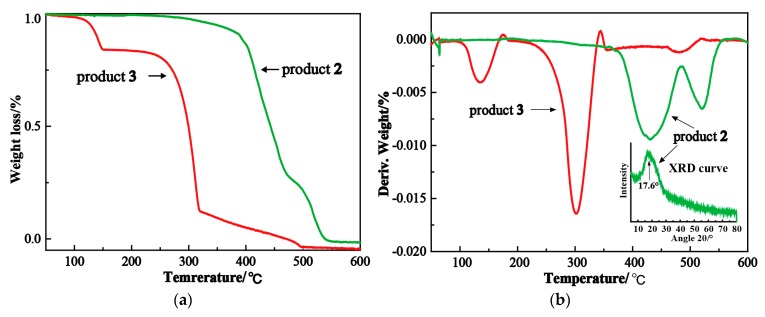
TG characterization of products **2** and **3**. (**a**) TG curves of products **2** and **3**. (**b**) DTG curves of products **2** and **3**.

The TG and DTG curves of products **2** and **3** are shown in Figure 3a,b, respectively.

The weight losses of poly(norbornene-(*N*-methyl)-phthalimide) occurred in the temperature ranges of 365–484 °C and 490–552 °C, respectively. Product **2** exhibited a sharp diffraction peak at 2θ = 17.6 ° (inset in Figure 3b), indicating its crystalline property. Therefore, the first weight loss might be attributed to the decomposition of the disordered region of poly(norbornene-(*N*-methyl)-phthalimide), and the other weight loss at the higher temperature was due to the decomposition of its crystalline region. The first weight loss temperature of 365–484 °C is higher than that of poly(norbornene-methylamine), possibly because the benzene ring on the side chain of poly(norbornene-(*N*-methyl)-phthalimide) is more stable than the amino group of poly(norbornene-methylamine). 

The first weight loss of poly(norbornene-methylamine) occurred in the temperature range of 97–174 °C, due to its highly active amino group that usually reduces the stability of polymer. The active –NH_2_ group in poly(norbornene-methylamine) might be attached to the double bond in the main chain to form bulky alkyl group by intramolecular cyclization. The interaction between –NH_2_ and the double bond was reported by Zvonimir et al. [29]. They found that the gas phase basicity of iminocyclopropene could be enhanced by the NH_2_ groups attached to the C=C double bond and subsequent substitution(s) by bulky alkyl group(s). The NH_2_ groups could release some of the lone pair electron density distribution, enabling a uniform distribution of the positive charge over the whole molecule. The intramolecular cyclization decreased the first weight loss temperature of poly(norbornene-methylamine) from 365–484 °C to 97–174 °C. Lv reported similar phenomenon in a thermally degradable aliphatic polyester bearing 2-aminoethanethiol -groups (P1) with polyester and amino-BOC (P1-BOC) [30,31]. They found the first weight loss of P1-BOC was initiated at 198.2 °C, due to the fact that the pyrolysis of the BOC group and P1 was unstable even at room temperature, because of the nucleophilicity of the pendent amino groups with a five-membered lactam formed by the intramolecular cyclization. Therefore, they concluded that polyesters bearing amino groups that caused larger steric hindrance and weaker nucleophilicity were more thermostable. Similarly, the phthalimide in poly(norbornene-(*N*-methyl)-phthalimide) can reduce the effect of amino groups on double bonds. The main chain of poly(norbornene-methylamine) was decomposed at 224–337 °C, resulting in a second weight loss.

These TG results of product **2** and [3] indicate that poly(norbornene-(*N*-methyl)-phthalimide) was successfully deprotected to afford the target product.

### 2.4. Optimization of Synthesise Condition for Product ***1***

The amino group of 5-norbornene-2-methylamine can be oxidized by other functional groups during the late reaction stage due to its strong reactivity, which may affect the activity of Grubbs catalyst. Therefore, it is necessary to protect amino groups before the ROMP reaction. To determine the optimal condition for the protection of amino groups, the yields of product **1** at different reaction times and temperatures were investigated.

**Figure 4:**
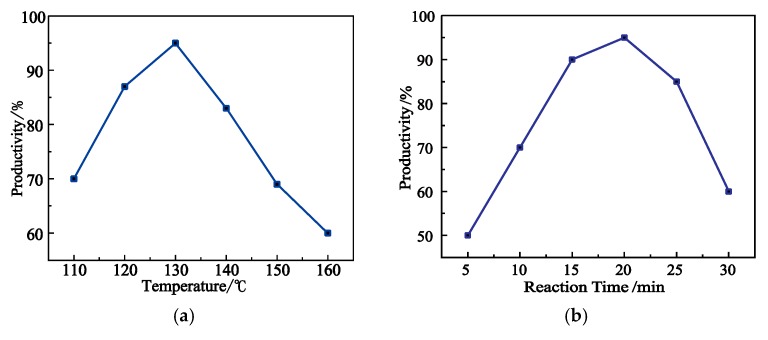
Yields of product **1** under different reaction conditions. (**a**) Effects of reaction temperature on the yield of product **1**. (**b**) Effects of reaction time on the yield of product **1** at 130 °C.

Figure 4a shows the variation in the yield of product **1** with reaction temperature at the reaction time of 20 min. The yield increased with the increase of reaction temperature, reached the maximum at 130 °C, and decreased as the reaction temperature further increased. This can be explained by the fact that the phthalic anhydride was melted at 130 °C, and thus completely reacted with 5-norbornene -2-methylamine, resulting in the highest yield. 5-norbornene-2-methylamine was evaporated at higher temperatures, which thus reduced the yield.

Figure 4b shows the yield changes of product **1** with reaction time at 130 °C. The yield increased with the prolongation of reaction time, reached the maximum at a reaction time of 20 min, and subsequently decreased, with the product becoming a brownish red due to the side reaction as the reaction time further prolonged. 

In summary, the synthesis condition of product **1** was optimized as: a reaction time of 20 min and a reaction temperature of 130 °C. The yield of product **1** reached 94.5% under the optimal synthesis conditions.

### 2.5. Molecular Weight and Polydispersity Index

**Figure 5:**
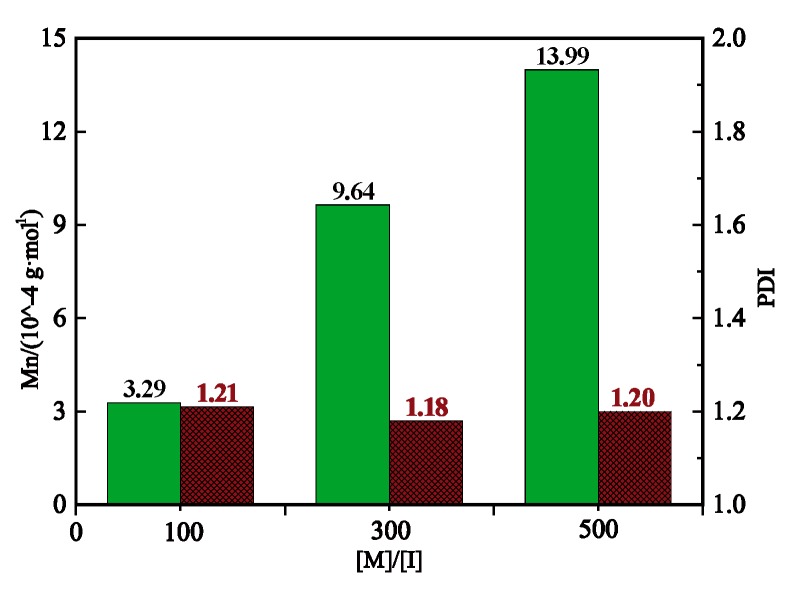
$\bar{Mn}$ and PDI of poly(norbornene-(*N*-methyl)-phthalimide) prepared by a 2 h ROMP at room temperature with different [M]/[I] ratios.

Figure 5 shows the effects of the molar ratio of monomer ([M]/[I]) on the molecular weight ($\bar{Mn}$) and polydispersity index (PDI) of poly(norbornene-(*N*-methyl)-phthalimide) deduced from the GPC results. The $\bar{Mn}$ increased linearly with [M]/[I] and exhibited a narrow PDI between 1.18 and 1.25. Therefore, the $\bar{Mn}$ of poly(norbornene-(*N*-methyl)-phthalimide) can be tuned by adjusting the [M]/[I] ratio. 

## 3. Materials and Methods

### 3.1. Materials

5-norbornene-2-methylamine (≥98%) was purchased from TCI Development Co., Ltd. (Shanghai, China). Di-*tert*-butyl dicarbonate and 2,6-di-*tert*-butyl paracresol were purchased from Sinopharm Chemical Reagent Co., Ltd. (Beijing, China). Phthalic anhydride was provided by Tianjin Xingfu Technology Development Co., Ltd. (Tianjing, China). Ethyl vinyl ether and the 2nd generation Hoveyda-Grubbs catalyst (≥97%) were purchased from Aladdin Industrial Co. (Shanghai, China). Hydrazine hydrate (80%) was purchased from Beijing Tongguang Fine Chemicals Company (Beijing, China). CH_2_Cl_2_ was dried with CaH_2_ and freshly distilled prior to use. 5-norbornene-2-methylamine was of gas chromatography grade and spectroscopy grade. Other reagents were of chemical grade.

### 3.2. Synthesis of 5-Norbornene-2-(N-methyl)-phthalimide (Product ***1***)

5-norbornene-2-methylamine (0.246 g) was added to 0.291 g phthalic anhydride and stirred under nitrogen at 130 °C for 20 min. The mixture was then added to 15 mL *n*-hexane, heated to dissolve, cool-crystallized, and dried to afford product **1**.

### 3.3. Synthesis of Poly(norbornene-(N-methyl)-phthalimide) (Product ***2***)

One milligram Grubbs II catalyst was dissolved in 1 mL CH_2_Cl_2_, vacuum aerated with nitrogen for 3 times, and stirred under nitrogen for 15 min. The catalyst solution was added to 3 mL product **1** (0.1 g) solution and stirred under nitrogen at room temperature for 2 h. To terminate the polymerization reaction, ethyl vinyl ether was added to the reaction solution and stirred for an additional 20 min. The final solution was poured into 400 mL of methanol to precipitate the polymer that was then dried to afford product **2**.

### 3.4. Synthesis of Poly(norbornene-methylamine) (Product ***3***)

Product **2** (0.2 g) was dissolved in 10 mL ethanol and refluxed with 2 mL of hydrazine hydrate and a certain amount of 2, 6-Di-*tert*-butyl-4-methylphenol under nitrogen at 100 °C for 8 h. The reaction solution was cooled to room temperature and precipitated with CH_2_Cl_2_. The precipitate was dried to afford product **3**.

### 3.5. Characterization

IR spectra were recorded on a Nicolet8700 FT-IR spectrometer that manufactured by Thermo Nicolet Co., (Madison, WI, USA) in the range of 4000–500 cm^−1^ at a resolution of 4 cm^−1^ using KBr pellets.

^1^H-NMR spectra were measured with a Bruker 500 MHz NMR spectrometer using TMS in DMSO, CDCl_3_ and CF_3_COOD as the internal standards for 5-norbornene-2-(*N*-methyl)-phthalimide, poly(norbornene-(*N*-methyl)-phthalimide) and poly(norbornene-methylamine), respectively.

Gel permeation chromatography (GPC) was conducted on a system containing a GPC detector (Waters Breeze HPLC, Waters Company, Framingham, MA, USA) and PSS Waters HR-3, HR-4 and HR-6 columns at 35 °C. Tetrahydrofuran (THF) was used as the mobile phase. 

Thermogravimetric analysis (TG) was performed on a DTG-60 TGA/DTA Analyzer (Shimadzu, Japan). The sample was heated from 30 °C to 600 °C at 10 °C/min under 50 mL/min nitrogen flow.

## 4. Conclusions

In the current study, poly(norbornene-methylamine) was prepared by ROMP for the first time. 5-norbornene-2-(*N*-methyl)-phthalimide was first prepared by the reaction between 5-norbornene-2-methylamine and phthalic anhydride with the highest yield of 94.5% achieved at 130 °C for 20 min. The ROMP reaction of 5-norbornene-2-(*N*-methyl)-phthalimide proceeded in the presence of Hoveyda-Grubbs 2nd catalyst to form poly(norbornene-(*N*-methyl)-phthalimide). The molecular weight ($\bar{Mn}$) of poly(norbornene-(*N*-methyl)-phthalimide) increased linearly with the increase of [M]/[I] ratio and exhibited a narrow PDI range of 1.18–1.25. Poly(norbornene-(*N*-methyl)-phthalimide was deprotected to afford the target polymer, poly(norbornene-methylamine). The products of each step were characterized with FTIR, ^1^H-NMR and TG. Our study provides a strategy for synthesizing chitosan like polymers bearing free –NH_2_ groups with controllable structures via ROMP.
